# A structural study of the interaction between the Dr haemagglutinin DraE and derivatives of chloramphenicol

**DOI:** 10.1107/S0907444909005113

**Published:** 2009-05-15

**Authors:** David M. Pettigrew, Pietro Roversi, Stephen G. Davies, Angela J. Russell, Susan M. Lea

**Affiliations:** a Sir William Dunn School of Pathology, University of Oxford, South Parks Road, Oxford OX1 3RE, England; b Department of Chemistry, Chemistry Research Laboratory, 12 Mansfield Road, Oxford OX1 3TA, England

**Keywords:** Dr adhesins, Dr haemagglutinin, DraE, chloramphenicol

## Abstract

Dr adhesins are expressed on the surface of uropathogenic and diffusely adherent strains of *Escherichia coli*. The major adhesin subunit (DraE/AfaE) of these organelles mediates attachment of the bacterium to the surface of the host cell and possibly intracellular invasion through its recognition of the complement regulator decay-accelerating factor (DAF) and/or members of the carcinoembryonic antigen (CEA) family. The adhesin subunit of the Dr haemagglutinin, a Dr-family member, additionally binds type IV collagen and is inhibited in all its receptor interactions by the antibiotic chloram­phenicol (CLM). In this study, previous structural work is built upon by reporting the X-ray structures of DraE bound to two chloramphenicol derivatives: chloramphenicol succinate (CLS) and bromamphenicol (BRM). The CLS structure demonstrates that acylation of the 3-hydroxyl group of CLM with succinyl does not significantly perturb the mode of binding, while the BRM structure implies that the binding pocket is able to accommodate bulkier substituents on the *N*-­acyl group. It is concluded that modifications of the 3-­hydroxyl group would generate a potent Dr haemagglutinin inhibitor that would not cause the toxic side effects that are associated with the normal bacteriostatic activity of CLM.

## Abstract 2

The structures of two Dr adhesin (DraE) complexes with chloramphenicol derivatives, namely chloramphenicol succinate and bromamphenicol, have been solved. The structures reveal important functional groups for small-molecule binding and imply possible modifications to the molecule that would permit a more wide-ranging interaction without the toxic side effects associated with chloramphenicol.

## 1. Introduction

Dr adhesins are thought to be important virulence factors for diffusely adherent (DAEC) and uropathogenic (UPEC) strains of *Escherichia coli*. Members of the Dr adhesin family include the Dr haemagglutinin, Dr-II, Afa-III and F1845 fimbriae (Servin, 2005 ▶). Estimates as to the frequency of Dr-family members in DAEC isolates range from 50% to 75% (Giron *et al.*, 1991 ▶). They are also the third most common group of colonization factors for UPEC behind type 1 and P pili (Nowicki *et al.*, 2001 ▶), with 25–50% of cystitis isolates from children and 30% of pyelonephritis isolates in pregnant women expressing Dr adhesins (Labigne-Roussel & Falkow, 1988 ▶). In addition, infection with Dr adhesin-positive UPEC is associated with an increased risk of a recurrent urinary-tract infection (Foxman *et al.*, 1995 ▶).

The Dr haemagglutinin, a member of the Dr adhesin family, can utilize up to four receptors to adhere to the host. The major adhesin subunit DraE recognizes decay-accelerating factor (DAF; Bernet-Camard *et al.*, 1996 ▶) and members of the carcinoembryonic antigen (CEA) family (Guignot *et al.*, 2000 ▶, 2009 ▶) and can trigger the bacterial mobilization of α_5_β_1_ integrin (Guignot *et al.*, 2009 ▶). DraE also binds type IV collagen (Westerlund *et al.*, 1989 ▶; Carnoy & Moseley, 1997 ▶). Together, these interactions are capable of orchestrating some of the host effects that are associated with the DAEC and UPEC pathotypes (reviewed by Servin, 2005 ▶).

The receptor interactions of DraE are inhibited by the antibiotic chloramphenicol (CLM). CLM inhibition of DraE was serendipitously discovered by Nowicki *et al.* (1988 ▶), who noticed that a CLM-contaminated reagent completely abolished DAF-mediated mannose-resistant haemagglutination (MRHA). CLM also inhibits the type IV collagen and CEA receptor interactions (Westerlund *et al.*, 1989 ▶; Korotkova, Cota *et al.*, 2006 ▶). In the years following the Nowicki group discovery, CLM was found to compare very effectively with other inhibitors of bacterial adhesion, its minimum inhibitory concentration (MIC) being 2 µ*M* compared with equivalent MICs for type 1 and P pili (carbohydrate) receptor analogues of between 400 and 500 µ*M* (Old, 1972 ▶; Svenson *et al.*, 1983 ▶). More recently, however, novel classes of carbohydrate derivatives have been developed with affinities in the nanomolar range specifically for type 1 or P pili (Bouckaert *et al.*, 2005 ▶; Ohlsson *et al.*, 2005 ▶). The present work represents a first ex­ploratory step towards CLM-based, less toxic and perhaps more specific inhibitors of DraE-mediated bacterial adhesion. For example, chloramphenicol succinate (CLS) inhibits DAF-mediated MRHA at similar levels to CLM (Nowicki *et al.*, 1988 ▶), although the stability of CLS to hydrolysis has not been characterized and the molecule is likely to be quickly metabolized to CLM *in vivo*.

The Dr haemagglutinin is the only CLM-sensitive Dr adhesin-family member (Le Bouguenec *et al.*, 1993 ▶). Our recently determined X-ray structure of the DraE–CLM com­plex has uncovered the molecular basis of the specificity of CLM for DraE (Pettigrew *et al.*, 2004 ▶). Binding was observed to be dependent on a shallow hydrophobic depression on the surface of DraE defined by residues Pro40–Pro43 on strand *B* and Ile111, Gly113 and Tyr115 on strand *E*. The structure of AfaE-III (Anderson *et al.*, 2004 ▶), a close relative of DraE with 98% sequence identity (three residue differences out of 140), demonstrates that the mutation of residue 88 from threonine (DraE) to methionine (AfaE-III) is sufficient to mask the binding pocket, thereby abolishing CLM sensitivity.

The conventional bacteriostatic activity of CLM arises from its ability to act as an inhibitor of the 50S prokaryotic ribosome (Moazed & Noller, 1987 ▶). In this context, the principal requirements for CLM activity are the 1,3-propanediol moiety and a *para*-substituted electron-withdrawing (nitro) group on the aromatic ring (Nagabhushan *et al.*, 1991 ▶). The structure of CLM complexed with the 50S ribosome of *Deinococcus radiodurans* has demonstrated that CLM inhibits protein biosynthesis in prokaryotes by binding to the peptidyl transferase active site (Schlunzen *et al.*, 2001 ▶). This interaction is particularly dependent on the burial of the nitrobenzene group and on hydrogen-bonding interactions between the primary hydroxyl group (3-OH) of CLM and ribosomal RNA. In contrast, our X-ray structure of the complex between DraE and CLM demonstrates that this CLM interaction is somewhat different in character, being more dependent on the hydrophobic burial of the dichloromethyl group of CLM rather than the aromatic ring or the hydroxyl groups (Pettigrew *et al.*, 2004 ▶). Since CLM is toxic in a small number of susceptible individuals, owing to its ability to traverse cell membranes and inhibit mitochondrial ribosomes (Kroon & Van den Bogert, 1983 ▶), we proposed that the DraE–CLM structure could provide a starting point for the design of an agent designed to inhibit Dr adhesion without the toxic side effects that are associated with the ‘normal’ bacteriostatic activity of CLM.

In the present study, a number of CLM derivatives were designed, cocrystallized with DraE and examined using X-ray crystallography in order to unambiguously determine which functional groups are important for DraE binding. We present the structures of chloramphenicol succinate and brom­amphenicol bound to DraE and use them to suggest possible modifications to the small molecule that could result in a safer inhibitor with a more wide-ranging action against other Dr adhesin-family members.

## 2. Materials and methods

### 2.1. Protein expression and purification

Recombinant DraE was expressed as an N-terminal hexahistidine fusion in *Escherichia coli* strain M15[pREP4] (Qiagen) and purified as described previously (Pettigrew *et al.*, 2004 ▶). Briefly, DraE was purified from the supernatant using nickel-affinity chromatography, followed by size-exclusion chromatography on an Superdex S75 Sepharose column (GE Life Sciences) to separate the trimeric form from small amounts of monomeric, dimeric and aggregated forms. The trimeric form was concentrated to 1.6 mg ml^−1^ and used in crystallization trials. Although the DraE trimer has previously been shown to be an artefact resulting from ‘domain swapping’ of strand *A*1 (rather than strand *G*, as observed in native Dr fimbriae), the chloramphenicol-binding site is intact in this form of the protein (Pettigrew *et al.*, 2004 ▶). This construct is amenable to crystallization in a number of different crystal forms, which makes it a convenient route to determining a number of CLM-derivative–DraE cocrystal structures.

### 2.2. Synthesis

**Figure 1:**
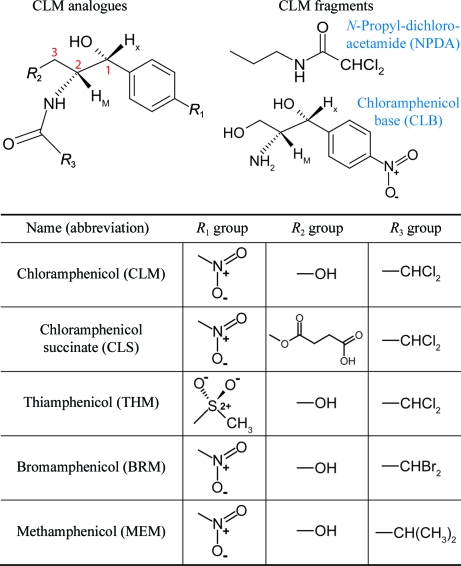
Chloramphenicol derivatives studied.

The various CLM derivatives and their abbreviations are summarized in Fig. 1 ▶. Chloramphenicol succinate (CLS), chloramphenicol base (CLB) and thiamphenicol (THM) were obtained from commercial sources (Sigma–Aldrich, UK). All the other analogues were synthesized as described below.

All reactions involving moisture-sensitive reagents were carried out in a nitrogen atmosphere using standard vacuum-line techniques and glassware that was flame-dried and cooled under nitrogen before use. The solvents were dried according to the procedure outlined by Grubbs and coworkers (Pangborn *et al.*, 1996 ▶). Water was purified using an Elix UV-10 system. All other solvents were used as supplied (analytical or HPLC grade) without prior purification. Thin-layer chromatography was performed on aluminium plates coated with 60F^254^ silica. The plates were visualized using UV light (254 nm), iodine, 1% aqueous KMnO_4_ or 10% ethanolic phosphomolybdic acid. Melting points were recorded on a Gallenkamp Hot Stage apparatus and are uncorrected. Optical rotations were recorded on a PerkinElmer 241 polarimeter with a water-jacketed 10 cm cell. Specific rotations are reported in 10^−1^ deg cm^2^ g^−1^ and concentrations (c) in grams per 100 ml. NMR spectra were recorded on Bruker Avance spectrometers in the deuterated solvent stated. The field was locked by external referencing to the relevant deuteron resonance.

#### 2.2.1. *N*-Propyl 2,2-dichloroacetamide (NPDA)

To a stirred solution of 1-aminopropane (0.70 ml, 8.5 mmol) in CH_2_Cl_2_ (30 ml) at 273 K was added a solution of dichloro­acetyl chloride (0.81 ml, 8.5 mmol) in CH_2_Cl_2_ (20 ml) dropwise. The resulting solution was stirred at 273 K for 1 h and then at room temperature (RT) for 2 h. The solution was washed successively with saturated aqueous NaHCO_3_ (10 ml), saturated aqueous NaCl (10 ml), dried (MgSO_4_) and the solvent removed with a vacuum pump (*in vacuo*). The crude product was recrystallized from ethyl acetate/pentane to yield the title compound as a white crystalline solid (1.16 g, 80%): melting point (m.p.) 321–323 K [literature melting point (lit. m.p.) 322–323 K; Matsumura *et al.*, 1976 ▶]; δ_H_ (400 MHz, methanol-*d*_4_) 0.96 (3H, t, *J* = 7.5 Hz, C*H*_3_), 1.53–1.62 (2H, m, C*H*_2_CH_3_), 3.22 (2H, t, *J* = 7.1 Hz, CONHC*H*_2_), 6.24 (1H, s, COC*H*Cl_2_).

#### 2.2.2. (1′*R*,2′*R*)-2,2-Dibromo-*N*-[1′,3′-dihydroxy-1′-(4′′-nitrophenyl)propan-2′-yl]acetamide (bromamphenicol/BRM)

To a stirred solution of dibromoacetic acid (0.21 ml, 2.3 mmol) in tetrahydrofuran (THF, 20 ml) under N_2_ at 273 K was added triethylamine (0.32 ml, 2.3 mmol) and then trimethyl­acetyl chloride (0.28 ml, 2.3 mmol) dropwise. The resulting suspension was stirred at 273 K for 1 h. The supernatant solution was decanted and added dropwise to a stirred solution of (1*R*,2*R*)-2-amino-1-(4′-nitrophenyl)propane-1,3-diol (490 mg, 2.3 mmol) at 273 K. The resulting solution was stirred at 273 K for 1 h and then RT for 2 h and was then quenched with saturated aqueous ammonium chloride (5 ml). The organic phase was separated and the aqueous phase was extracted with ethyl acetate (2 × 20 ml). The combined organic extracts were washed successively with saturated aqueous NaHCO_3_ (10 ml) and saturated aqueous NaCl (10 ml), dried (MgSO_4_) and the solvent was removed *in vacuo*. The crude product was purified by recrystallization from methanol/diethyl ether to give the title compound as a white crystalline solid (590 mg, 62%): m.p. 321–323 (lit. m.p. 325–326 K; Moersch, 1955 ▶); [α]_D_^22^ +22.4 (c 1.0, EtOH) {lit. [α]_D_^25^ +19.6 (c 1.0, EtOH) (Moersch, 1955 ▶)}; δ_H_ (400 MHz, methanol-*d*_4_) 3.62 [1H, dd, *J* = 10.8, 6.0 Hz, C(3′)*H*_*2A*_], 3.83 [1H, dd, *J* = 10.8, 7.4 Hz, C(3′)*H*_*2B*_], 4.12–4.16 [1H, m, C(2′)*H*], 5.19 [1H, d, *J* = 2.3 Hz, C(1′)*H*], 6.19 [1H, s, C(1)*H*], 7.67 [2H, d, *J* = 8.4 Hz, Ar(2′′)*H* and Ar(6′′)*H*], 8.20 [2H, d, *J* = 8.4 Hz, Ar(3′′)*H* and Ar(5′′)*H*].

#### 2.2.3. (1′*R*,2′*R*)-*N*-[1′,3′-Dihydroxy-1′-(4′′-nitrophenyl)pro­pan-2′-yl]isobutyramide (methamphenicol/MEM)

To a stirred solution of (1*R*,2*R*)-2-amino-1-(4′-nitrophenyl)propane-1,3-diol (500 mg, 2.4 mmol) in THF (20 ml) at 273 K was added triethylamine (0.33 ml, 2.4 mmol) and then a solution of iso­butyryl chloride (0.25 ml, 2.4 mmol) in THF (20 ml) dropwise. The resulting solution was stirred at 273 K for 1 h and then RT for 2 h. The solvent was removed *in vacuo* and the residue was taken up in ethyl acetate (30 ml). The solution was washed successively with 10% aqueous HCl (10 ml), saturated aqueous NaHCO_3_ (10 ml), saturated aqueous NaCl (10 ml), dried (MgSO_4_) and the solvent was removed *in vacuo*. The crude product was recrystallized from ethyl acetate/pentane to yield the title compound as a white crystalline solid (450 mg, 68%): m.p. 301–303 K (lit. m.p. 304.5–306 K; Shirahata *et al.*, 1972 ▶); [α]_D_^22^ −56.4 (c 1.1, EtOH) {lit. [α]_D_^25^ −61 (c 1.0, EtOH) (Shirahata *et al.*, 1972 ▶)}; δ_H_ (400 MHz, methanol-*d*_4_) 0.89 (3H, d, *J* = 6.9 Hz, CH*Me*_2_), 1.01 (3H, d, *J* = 6.8 Hz, CH*Me*_2_), 2.39–2.48 (1H, m, C*H*Me_2_), 3.61 [1H, dd, *J* = 10.8, 5.9 Hz, C(3′)*H*_*2A*_], 3.81 [1H, dd, *J* = 10.8, 7.4 Hz, C(3′)*H*_*2B*_], 4.19–4.23 [1H, m, C(2′)*H*], 5.18 [1H, d, *J* = 2.6 Hz, C(1′)*H*], 7.66 [2H, d, *J* = 8.5 Hz, Ar(2′′)*H* and Ar(6′′)*H*], 8.21 [2H, d, *J* = 8.5 Hz, Ar(3′′)*H* and Ar(5′′)*H*].

### 2.3. Crystallization

Cocrystallization of a number of CLM-analogue–DraE complexes was attempted using a crystallization robot (Tecan, Zurich, Switzerland) to generate a large number of ammonium sulfate concentration *versus* small-molecule concentration grid screens at pH 7.0 (0.1 *M* Na HEPES).

DraE–CLS cocrystals in space group *P*3 were obtained in 2 *M* ammonium sulfate, 0.1–20 m*M* CLS and 0.1 *M* Na HEPES pH 7.0. One unique crystal crystallized in space group *C*2 in a drop containing 10 m*M* CLS but could not be reproduced. Owing to the low solubility of bromamphenicol (BRM) and thi­amphenicol (THM), soaks of these small molecules into pre­formed CLS cocrystals were also attempted. CLS cocrystals were transferred to a mother-liquor solution with­out CLS and left for 15–20 min to reduce the occupancy of the small molecule in the binding site. Longer backsoaking, which, in principle, would have been needed to soak the ligand out (Collins *et al.*, 2007 ▶), led to visible damage to the crystals and could not be afforded. The crystal was then placed in a mother-liquor solution saturated with either BRM or THM and soaked for a further 15 min.

### 2.4. Data collection and processing

X-ray diffraction data were collected in-house and on beamline ID-29 at the European Synchrotron Radiation Facility (ESRF). Crystals were cryoprotected in 25% ethylene glycol plus mother liquor or soaking solution before being mounted in a fibre loop and flash-frozen at 100 K. The data-reduction and refinement statistics are summarized in Table 1 ▶. Two crystal forms were obtained for successful CLM-derivative–DraE cocrystals: trigonal (*P*3) and monoclinic (*C*2). Within *MOSFLM* (Collaborative Computational Project, Number 4, 1994 ▶), initial autoindexing solutions based on two sets of five diffraction images (ϕ = 90° apart) were used to screen for successful small-molecule-bound derivatives. Crystals that initial indexing revealed as belonging to the orthorhombic polymorph deposited as PDB entry 1ut1 (space group *P*2_1_2_1_2_1_, unit-cell parameters *a* = 68.88, *b* = 108.51, *c* = 119.62 Å) were immediately discarded as packing interactions make the CLM-binding site in­accessible in this crystal form; these crystals must therefore form by favouring the subunit–subunit interaction over the subunit–ligand inter­action and are useless for the present study. Prior to the start of the DraE–BRM data collection, an X-ray fluorescence scan was taken to locate the bromine peak absorption wavelength (0.91947 Å). Data were collected at this wavelength to maximize the anomalous difference signal from the Br atoms of BRM. Data reduction was performed using *SCALA* (Evans, 2006 ▶). The Friedel pairs were kept separate for the BRM data set and were merged for the others.

### 2.5. Structure solution and refinement

#### 2.5.1. *P*3 data sets

Refinement of the CLM-derivative data sets in space group *P*3 (CLS, BRM and THM) were performed using the program *autoBUSTER* (Vonrhein *et al.*, 2006 ▶). As the unit-cell parameters (*a* = *b* = 119.6, *c* = 57.8 Å) and symmetry were those of our previously determined crystal structure of the DraE–CLM complex (space group *P*3; *a* = *b* = 119.0, *c* = 57.4 Å; PDB code 1usq), this model, minus CLM, was used as a starting point for refinement. The binding site was excluded from the automatic addition of waters. The small-molecule conformation was then interpreted from an *F*_o_ − *F*_c_ map after all the other density had been explained. For each CLM derivative, the program *CORINA* (Sadowski *et al.*, 1994 ▶) was used to generate a coordinate file with appropriate bond lengths and angles. The program *XPLO*2*D* (Kleywegt & Jones, 1997 ▶) was used to generate a TNT stereochemical restraints dictionary from the resulting co­ordinate file. Soft noncrystallographic symmetry (NCS) restraints were then used in refining the atomic positions and temperature factors of the six NCS-related copies of the CLM derivative.

For the CLS–DraE data set in space group *P*3, an NCS-averaged 2*F*_o_ − *F*_c_ density map was produced after the CLS molecule had been built and refined in the absence of the succinate tail, since the succinate moiety was not visible in the initial difference map. The coordinates of the small molecule and the binding site (comprised of residues 37–45, 84–88 and 110–116) were used to define a 5 Å radius mask around the atoms using the program *NCSMASK* (Collaborative Com­putational Project 4, Number 4, 1994 ▶). The NCS operators and the mask were used as input for the program *MAPROT* (Collaborative Computational Project 4, Number 4, 1994 ▶) to produce the NCS-averaged 2*F*_o_ − *F*_c_ density map. Three conformers for the succinate tail, each at an occupancy of 0.3, were built. 15 cycles of manual (NCS-restrained) *BUSTER-TNT* refinement with all six copies of CLS in place generated three energy-minimized succinate conformers.

For the BRM data set in space group *P*3, an anomalous difference map was calculated using the phases from the final refined model in the absence of small molecule. Inspection of this map unambiguously confirmed that the small-molecule density was a superposition of CLS and BRM. After the initial placement of CLS (minus the succinate tail) and BRM into the *F*_o_ − *F*_c_ map (each at 50% occupancy), an occupancy refinement of the small molecules determined the relative composition of each binding site. The small-molecule parameters were allowed to refine independently of NCS restraints as each binding site had a different occupancy.

#### 2.5.2. *C*2 data set

Initial phases for the CLS data in space group *C*2 were determined by searching with the DraE–CLM trimer in *MOLREP*, again with the CLM molecules omitted. 12 cycles of (monomer) rigid-body refinement were run using *BUSTER-TNT* (Blanc *et al.*, 2004 ▶) with no nonbonded restraints. Next, *autoBUSTER* was used to refine the model with soft NCS restraints and to add waters. After four rounds of rebuilding and refinement using *XtalView*/*Xfit* and *BUSTER-TNT*, respectively, the *F*_o_ − *F*_c_ map was inspected to confirm the presence of CLS in the binding site.

## 3. Results

Previously, we reported the structure of DraE bound to CLM (Pettigrew *et al.*, 2004 ▶). In this structure, CLM binding was observed to be focused on the chlorine ‘tail’ rather than the nitrobenzene ‘head’ of the molecule. The presence of CLM in the crystallization mother liquor results in a change of space group from the apo orthorhombic (*P*2_1_2_1_2_1_) crystal form to the ‘drug-bound’ trigonal (*P*3) crystal forms. This is a consequence of the binding site being involved in a crystal contact in the orthorhombic crystal form (see §[Sec sec3.4]3.4 below for details of this crystal contact).

In the present work, a number of CLM analogues were tested in order to determine which functional groups of CLM are important for DraE interaction (Fig. 1 ▶). Cocrystals were very difficult to obtain for most of the small molecules owing to their extremely poor aqueous solubility, while soaks of the required CLM derivative into pre-formed CLM/CLS cocrystals were complicated by residual CLM/CLS in the binding site. Therefore, this route to structure determination was only attempted for small molecules with easily identifiable functional groups (such as the anomalous scatterers of BRM or the *para*-substituted methyl sulfone group of THM).

All the cocrystals described in this work belong to the *P*3 form (with the exception of a single irreproducible *C*2 DraE–CLS crystal that grew in 10 m*M* CLS); both *C*2 and *P*3 crystals contain DraE trimers, as did the apo *P*2_1_2_1_2_1_ crystals (and the related AfaE-III cubic and trigonal crystals). The physio­logical intersubunit interactions that give rise to the adhesin fibres *in vivo* are those described in the published NMR study of DraE (Anderson *et al.*, 2004 ▶), while the crystals contain strand-swapped trimers (Pettigrew *et al.*, 2004 ▶): as explained in the latter study, the trimers in these crystals arise from inter-subunit strand swapping and have no physiological relevance, but the nonphysiological intersubunit strand swapping does not affect the CLM ligand binding as it occurs at a site distal with respect to the site of fibre and trimer formation. This was proved by the NMR chemical shift mapping upon CLM titration, which agreed with the CLM-binding site of the crystal structures and was conducted on the donor-strand exchanged DraE construct that does not give rise to trimers (Pettigrew *et al.*, 2004 ▶). Also entirely crystal-related and therefore of no physiological relevance is the intertrimer noncrystallographic symmetry that describes the packing of the trimers; for this reason, we do not describe the packing nor the noncrystallographic symmetry in detail (suffice here to say that in both crystal forms the trimers are arranged in slabs laying in the *ab* planes and are stacked along the *c* direction, both the *a* and *c* unit-cell axes being of similar length in the two forms; see Table 1 ▶).

### 3.1. *N*-Propyldichloroacetamide and chloramphenicol base

The observed mode of CLM binding to DraE suggested that the interaction is focused on the burial of the chlorines. In order to investigate this in more detail, two CLM fragments consisting of the aliphatic ‘tail’ (*N*-propyldichloroacetamide; NPDA) and the aromatic ‘head’ (chloramphenicol base; CLB) groups were studied (Fig. 1 ▶). Unfortunately, NPDA abolished the crystallization of DraE at small-molecule concentrations greater than 0.1 m*M* and only the apo crystals were obtained for lower concentrations. This could imply that NPDA bound in the CLM-binding pocket. In this scenario, crystallization would be abolished by the inability of the small molecule to stabilize the appropriate crystal contacts in the trigonal crystal form. Alternatively, NPDA may have bound nonspecifically to the protein, thereby perturbing crystal contacts elsewhere. Crystallization mother liquors containing CLB at concentrations of up to 1 m*M* generated the apo orthorhombic crystal form. Since the concentration of CLB did not appear to influence the crystallization of the apo crystal form, we con­cluded that CLB did not interact specifically with the CLM-binding site or nonspecifically with a portion of DraE that was involved in a crystal contact. This hypothesis is supported by the observation that CLB is unable to inhibit the haemagglutination of erythrocytes (Nowicki *et al.*, 1988 ▶).

### 3.2. Chloramphenicol succinate

**Figure 2:**
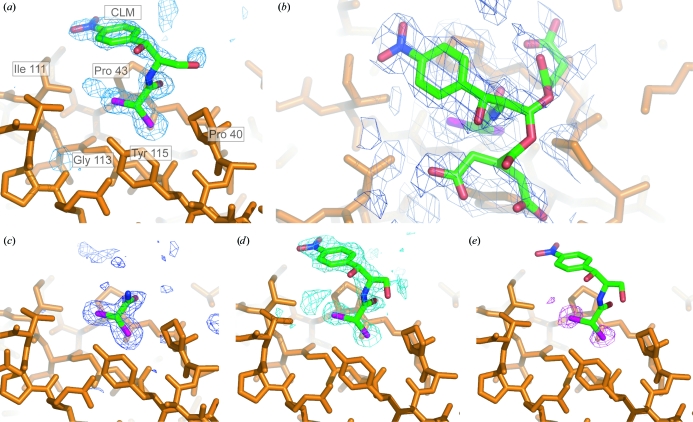
The physiological ligand-binding site. (*a*, *b*, *c*) Maps and models for chloramphenicol succinate: the refined model of CLS is shown [with the succinate tail ini (*b*) and without it in (*a*)]. (*a*) Unbiased *F*_o_ − *F*_c_ map (data set DraE–CLS.2) contoured at 2.5σ showing the CLS density in the hydrophobic pocket (*P*3 crystal form). (*b*) The NCS-averaged 2*F*_o_ − *F*_c_ electron density in the binding pocket (*P*3 crystal form, averaged over all six copies of the density in the ASU) reveals three possible lowest energy conformers for the succinate tail. (*c*) Unbiased *F*_o_ − *F*_c_ map (blue mesh, data set DraE–CLS.1) contoured at 2.3σ, showing that in the *C*2 crystal form the aromatic head group and the succinate tail are disordered. (*d*) The same view as in (*a*) for the BRM derivative, with the unbiased *F*_o_ − *F*_c_ difference map (contoured at 2.2σ) shown as a blue mesh. (*e*) Anomalous difference map (purple mesh) for the BRM derivative calculated in the resolution range 25–1.9 Å using phases from the protein model only (contoured at 3.5σ). It clearly reveals the presence of two bromines in the CLM-binding site. The final refined BRM model (20% occupancy) is also shown. The final refined CLS model is not shown for reasons of clarity, but it adopts an identical conformation. Figures were rendered in *PyMOL* (DeLano, 2004 ▶).

In order to establish whether substitution at the primary hydroxyl of CLM influences the binding of the small molecule to DraE, the structure of a DraE–CLS complex was determined. CLS cocrystals in space group *P*3 were reproducibly obtained using small-molecule concentrations between 1 and 15 m*M*. In this crystal form, the unbiased *F*_o_ − *F*_c_ difference map showed CLM-like density in the region of the physiological binding site (Fig. 2 ▶ *a*). The aliphatic ‘tail’ and the nitro-benzene ‘head’ group were traced within this electron density. Positional and temperature (*B*) factor refinement confirmed that CLS was present in the binding site at full occupancy, with an average *B* factor of 32 Å^2^. No difference density was resolved for the succinate tail, implying that these atoms adopted a number of alternate conformers that were not resolved at 1.9 Å resolution. However, an NCS-averaged 2*F*_o_ − *F*_c_ map revealed weak density for three possible low-energy succinate conformers (Fig. 2 ▶ *b*). With each conformer set to an occupancy of 30%, the *B* factors refined to an average value of 49 Å^2^ for the succinate atoms, compared with an average value of 40 Å^2^ for the rest of the small molecule.

A unique CLS cocrystal in space group *C*2 was obtained at a small-molecule concentration of 10 m*M* and could not be reproduced. In this crystal form, the unbiased *F*_o_ − *F*_c_ map revealed density for the dichloroacetyl moiety of CLS in the physiological binding site (Fig. 2 ▶ *c*). However, even after temperature-factor and occupancy refinement with the di­chloroacetyl group in place, density for the nitrobenzene head group was not visible in any of the NCS-related binding sites. Ordering of the whole ligand in the *P*3 forms is helped by crystal contacts from a neighbouring protein molecule (Gln47 and Leu49) to the ligand atoms C_7_ and C_8_ (in the phenyl ring) and one of the O atoms on the nitro moiety (the atom labelled O_9B_); this crystal contact is missing in the C2 form and the ‘head group’ of CLS adopted a number of conformations that were not traceable. Overall, the CLS–DraE complex structures imply that (i) substitutions at the primary hydroxyl do not significantly affect the mode of CLM binding and (ii) the nitrobenzene group is not required to bind DraE.

### 3.3. Bromamphenicol and methamphenicol

To investigate the contribution of the aliphatic tail to the small-molecule interaction, attempts were made to cocrystallize DraE with methamphenicol (MEM; less hydrophobic than CLM) and BRM (more hydrophobic than CLM) (Fig. 1 ▶). MEM was tested at comparable concentrations to CLM (up to 1 m*M*). Despite this, only the apo crystal form was obtained. Therefore, the reduced size and hydrophobicity of the acetyl group of MEM relative to the dichloroacetyl group of CLM significantly reduced or abolished the DraE–small molecule interaction. BRM was nearly insoluble and even saturated BRM mother liquors were unable to produce anything other than the apo crystal form. At such negligible BRM concentrations it was impossible to reach any conclusions about the relative affinities of BRM and CLM for DraE.

BRM soaks into CLS cocrystals were performed and the anomalous signal of the bromines was exploited in order to unambiguously determine the location of the small molecule. The fluorescence scan showed a clear absorption edge in the region of the *K* absorption edge of bromine (data not shown). This confirmed that some BRM had been dissolved despite the low solubility of the molecule. The anomalous difference map revealed two peaks in the density at a position in the physiological binding site that was previously seen to be occupied by the chlorines of CLM/CLS (Fig. 2 ▶ *e*). The *f*′′ value for chlorine is low at the wavelength used in the experiment (0.26 compared with 3.82 for bromine at 0.91947 Å). Hence, the anomalous difference peaks in the binding site must arise from the presence of the bromines of BRM. In addition, the unbiased *F*_o_ − *F*_c_ difference map showed CLM-like density in the same region (Fig. 2 ▶ *d*). The entire BRM molecule was traced within this map. However, BRM did not refine at full occupancy, as strong negative residual peaks were observed in the region of the two bromines. After occupancy refinement of BRM, strong positive residuals were observed for all the small-molecule atoms except for the bromines. Therefore, the binding pocket contained a superposition of CLS and BRM in identical orientations. Repeated rounds of alternating occupancy and positional refinements revealed that BRM and CLS were present at between 20–30% and 50–70% occupancy, respectively, in the six NCS-related copies of the binding site. This structure demonstrated that the binding pocket of DraE can accommodate larger chemical groups.

**Figure 3:**
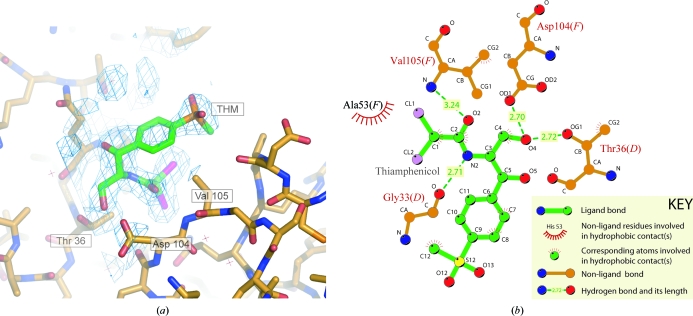
The nonphysiological THM-binding site. (*a*) The 2*F*_o_ − *F*_c_ map contoured at 1.3σ (calculated after refinement of the entire THM molecule) in the region of the nonphysiological binding site clearly shows the tetrahedral density of the sulfonyl group. (*b*) *LIGPLOT* (Wallace *et al.*, 1995 ▶) representation of THM at the same site. The number of each residue involved is given, together with the chain designation of the DraE subunit it originates from (*D* or *F*). An identical THM-binding site is also observed between copies *B* and *C*.

The anomalous difference map revealed two bromines at a secondary BRM-binding site which was defined by the interface between two copies of DraE. The small molecule bridged a crystal contact, but the area of contact between molecules is too small for it to be of significance (300 Å^2^; 3.8% of the monomer’s surface); this second binding site was therefore assumed to be an artefact of crystal packing. Moreover, CLM inhibition has been mapped to the area of surface close to the main binding site (Pettigrew *et al.*, 2004 ▶); henceforth, this site is referred to as the ‘nonphysiological’ binding site. The 2*F*_o_ − *F*_c_ and unbiased *F*_o_ − *F*_c_ maps in this region showed electron density corresponding to the dibromo­acetyl moiety of BRM (data not shown). Difference density for the 1,3-propanediol moiety was only resolved after refinement of the dibromoacetyl group. Even after refinement with the aliphatic ‘tail’ in place, the aromatic ‘head’ group could not be resolved in the 2*F*_o_ − *F*_c_ map or the *F*_o_ − *F*_c_ map. This implied that the aromatic group adopted a number of alternate conformers that could not be resolved at 1.9 Å resolution. The average temperature factor of the BRM fragment was 33 Å^2^, suggesting that the nonphysiological BRM-binding site was fully occupied. As an entire THM molecule was found in an identical orientation in the same nonphysiological binding site, the relevant protein–small molecule contacts are discussed below. Fig. 3 ▶ illustrates this second binding site and the orientation of the ligand in it.

### 3.4. Thiamphenicol

Preparation of THM–DraE cocrystals was attempted in order to determine whether *para* substitutions of the nitro group affected the interaction with DraE. The aromatic ‘head’ group of THM comprises a *para*-substituted methyl phenyl sulfone moiety (Fig. 1 ▶). Soaks of THM into CLS cocrystals were attempted based on the assumption that the tetrahedral methyl sulfone group of THM could be distinguished from the planar nitro group of CLS in the electron density.

Inspection of the unbiased *F*_o_ − *F*_c_ difference density in the region of the physiological binding pocket revealed a planar rather than a tetrahedral group at the *para* position of the benzene ring (data not shown). Indeed, CLS refined successfully at full occupancy and no residual peaks were observed in the difference map. Therefore, we concluded that THM was unable to enter the CLM-binding pocket of DraE in the *P*3 crystal form. Steric interference between the protein and the methylsulfonyl group of THM is the most likely reason for this.

As mentioned above, THM occupied the same nonphysiological binding site as BRM. 2*F*_o_ − *F*_c_ and *F*_o_ − *F*_c_ maps were calculated using phases from the protein, the CLS models in the physiological binding site and the dichloroacetyl group of THM in the nonphysiological binding site. The *para*-substituted tetrahedral moiety of THM was clearly observed in the nonphysiological binding site in both density maps. The entire THM molecule was traced within this difference density and no negative peaks were observed in the difference map after refinement with 100% THM occupancy (Fig. 3 ▶). The average *B* factor (33 Å^2^) was comparable to the average nonprotein *B* factor (27 Å^2^). Therefore, the two nonphysiological binding sites in the ASU were fully occupied.

The principal contacts between THM and the protein are given in Fig. 3 ▶. The two THM molecules in the ASU adopt a similar conformation to CLM in the physio­logical binding site and are positioned at the interface between two subunit copies. In Fig. 3 ▶ this is illustrated by subunits *D* and *F* (in the crystal the same binding site exists between the subunits labelled *B* and *C*). This predominately electrostatic interaction is mediated by residues within the loop between the first and third strand on one subunit (labelled *D* in Fig. 3 ▶) and by residues within a loop between the sixth and ninth β-strand on the other (labelled *F*). In more detail, hydrogen bonds are formed between the carbonyl of THM and the main-chain amide of Val105, the primary hydroxyl group OH(3) of THM forms hydrogen bonds to the side-chain atoms of Asp104 on one subunit (chains *F* and *C*) and Thr36 on the other (chains *D* and *B*), and a hydrogen bond occurs between the main-chain carbonyl of Gly33 and the amide within the aliphatic tail of THM. Van der Waals contacts are also formed between atoms of the aliphatic tail and residues within both DraE subunits. Only minor van der Waals contacts are observed between the *para*-substituted methyl phenyl sulfone moiety and the protein.

## 4. Discussion

There is an urgent need for novel therapeutics to combat the emergence of antibiotic-resistant strains of pathogenic bacteria (Breithaupt, 1999 ▶). An attractive approach is the use of agents that interfere with the ability of the bacterium to adhere to the host. As adhesion is an absolute requirement for successful colonization, prevention of adhesion at an early stage should prevent infection. Furthermore, since anti-adhesive agents do not kill or inhibit bacterial growth, it is expected that strains which are resistant to these agents will emerge at a much lower rate than those which are resistant to antibiotics (Ofek *et al.*, 2003 ▶). Studies on anti-adhesives for UPEC have focused on the use of carbohydrate-receptor analogues, which aim to competitively inhibit bacterial adhesion *via* the lectin domains of type 1 and P pili (reviewed by Sharon, 2006 ▶). As UPEC strains express more than one type of adhesin (Hacker, 1992 ▶), any therapeutic cocktail against UTIs must include an effective agent against Dr adhesins. One lead candidate is the antibiotic chloramphenicol (CLM), which abolishes the adhesion of the Dr haemagglutinin DraE to DAF, CEA and type IV collagen (Nowicki *et al.*, 1988 ▶; Westerlund *et al.*, 1989 ▶). All the derivatives of CLM studied here are expected to inhibit fimbrial adhesion in the same way as CLM does, given that they bind at the same site and that CLM binding at that site has previously been shown to disturb receptor adhesion (Pettigrew *et al.*, 2004 ▶).

### 4.1. Substitution at the primary hydroxyl of CLM does not perturb binding

The CLS structure in space group *P*3 proves that substitutions at the primary hydroxyl 3-OH do not affect the mode of CLM binding to DraE. In contrast, the 3-hydroxyl group is essential for ribosome inhibition; both chloramphenicol phosphotransferase and chloramphenicol acetyltransferase abolish the bacteriostatic activity of CLM by phosphorylation (Mosher *et al.*, 1995 ▶) and acetylation (Murray & Shaw, 1997 ▶), respectively, at this position. Therefore, we hoped that increased DraE specificity could be gained from substitution at the primary hydroxyl group; unfortunately, the CLS succinate in this study did not fit the surface with a single conformation and alternative substituents to the succinyl moiety will need exploring. In addition, the disordered nitrobenzene group in the DraE–CLS structure in space group *C*2 implies that the interaction with DraE is focused on the burial of the chlorines rather than the benzene ring. This raises the possibility that the nitrobenzene group is not required for DAF inhibition. Conversely, in the CLM–ribosome complexes the interaction is focused on the burial of the benzene ring and not the chlorines (Hansen *et al.*, 2003 ▶; Schlunzen *et al.*, 2001 ▶). DraE specificity could therefore be gained by modifying the aromatic ring or by dispensing with it altogether. However, although the nitrobenzene group is not required to bind to DraE, it may be important for inhibition. Therefore, functional studies are necessary to determine the precise requirements for drug inhibition with regard to the aromatic ring.

### 4.2. The CLM-binding pocket is able to accommodate bulkier substituents

The BRM structure demonstrates that the CLM-binding pocket of DraE is able to accommodate larger substituents on the *N*-acyl group. This raises the possibility that a more hydrophobic molecule could be used to displace the Met88 side chain from the binding pocket of AfaE-III, thereby providing a means of inhibiting Afa-III and the Dr haem­agglutinin. In this respect, the structure of an AfaE-III–BRM complex would be highly informative. However, our attempts to resolve this have so far been unsuccessful.

### 4.3. Possible mechanisms of chloramphenicol inhibition

**Figure 4:**
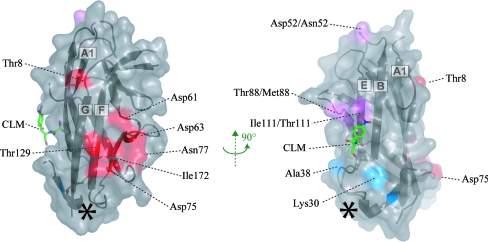
Receptor-binding sites on DraE. Solvent-accessible surface representation of a single DraE subunit, together with the location of CLM. Residues which have been reported to be important for DAF (red), collagen (blue and purple) and CLM (purple) binding are coloured (Van Loy *et al.*, 2002 ▶; Carnoy & Moseley, 1997 ▶). Note: Asp52 has not been implicated in collagen binding. The asterisk marks the site of the secondary nonphysiological THM and BRM binding.

A major obstacle to the design of an effective Dr adhesin inhibitor is our lack of understanding of the mechanism of DAF inhibition. CLM binds to a region of DraE that is involved in binding to CEA (Korotkova, Cota *et al.*, 2006 ▶) and possibly type IV collagen (Carnoy & Moseley, 1997 ▶), suggesting that CLM acts as a competitive inhibitor of both these receptor interactions. However, the attenuation of DAF binding is more puzzling. Mutagenesis (Van Loy *et al.*, 2002 ▶), surface plasmon resonance (Korotkova, Le Trong *et al.*, 2006 ▶) and NMR chemical shift mapping (Anderson *et al.*, 2004 ▶) studies have enabled the primary DAF-binding site to be identified as a negatively charged depression on a surface of DraE that is remote from the CLM-binding site (Fig. 4 ▶). Therefore, CLM must act as a noncompetitive inhibitor of DAF binding. This raises two possible mechanisms for DAF inhibition by CLM: either the small molecule disturbs the binding site indirectly and pre­vents rigid-body association or it prevents the conformational equilibrium of DraE from being pulled towards the DAF-bound state. The former possibility can be discounted, since CLM creates only local perturbations that do not involve residues implicated in DAF binding (Pettigrew *et al.*, 2004 ▶). The latter possibility is supported by the observation that DAF induces structural perturbations throughout the entire AfaE-III–DSC molecule (Anderson *et al.*, 2004 ▶). Therefore, CLM may lock DraE in its isolated solution state, thereby preventing it from undergoing the ‘induced-fit’ modifications required for DAF interaction. The structure of DraE bound to DAF would potentially offer new insights into how CLM inhibits noncompetitively. This would greatly facilitate our search for a more potent and specific Dr adhesin inhibitor.

## Supplementary Material

PDB reference: DraE–CLS, space group *C*2, 2w5p, r2w5psf

PDB reference: DraE–CLS, space group *P*3, 2jkn, r2jknsf

PDB reference: DraE–BRM, 2jkl, r2jklsf

PDB reference: DraE–THM, 2jkj, r2jkjsf

## Figures and Tables

**Table 1:** Data-reduction and refinement statistics Values in parentheses are for the highest resolution shell.

	DraE–CLS.1	DraE–CLS.2	DraE–BRM	DraE–THM
PDB code	2w5p	2jkn	2jkl	2jkj
Data statistics				
Beamline	ID14-3 ESRF	In-house	ID29 ESRF	In-house
Space group	*C*2	*P*3	*P*3	*P*3
Unit-cell parameters (Å, °)	*a* = 118.9, *b* = 68.6, *c* = 62.1, β = 111	*a* = *b* = 119.3, *c* = 57.7	*a* = *b* = 119.6, *c* = 57.8	*a* = *b* = 119.6, *c* = 57.7
Resolution (Å)	59–1.9 (2.0–1.9)	30–1.9 (2.0–1.9)	25–1.9 (2.0–1.9)	25–2.3 (2.4–2.3)
No. of unique reflections	33359	60758	72802	43763
Redundancy	4.1	3.3	6.7	4.3
Completeness (%)	96.8 (78.1)	98.2 (97.7)	100 (100)	100 (99.9)
Anomalous completeness	N/A	N/A	99.9 (100)	N/A
*R*_merge_	0.10 (0.38)	0.07 (0.21)	0.10 (0.24)	0.10 (0.33)
*R*_anom_	N/A	N/A	0.04 (0.11)	N/A
Average *I*/σ(*I*)	6.0 (2.0)	4.6 (2.1)	5.2 (3.0)	8.9 (2.2)
No. of molecules in ASU	3	6	6	6
Refinement statistics				
*R* (%)	20.4 (21.0)	19.0 (19.3)	17.9 (18.1)	24.9 (27.8)
*R*_free_ (%)	23.6 (24.5)	21.2 (22.3)	20.0 (19.7)	27.3 (30.7)
No. of residues/atoms in model				
No. of protein atoms	3211	6314	6323	6289
No. of nonprotein atoms	393	972	1095	391
Mean *B*, protein (Å^2^)	26	22	16	15
Mean *B*, nonprotein [mean *B* small molecule] (Å^2^)	37 [33]	36 [45†]	32 [35]	27 [32]
No. of sulfates/ethylene glycols/small molecules	3/0/6	12/12/6	14/11/8	15/0/8
Geometry				
R.m.s.d. bonds (Å)	0.006	0.009	0.004	0.003
R.m.s.d. angles (°)	0.8	0.9	0.8	0.7
Residues in				
Most favoured regions (%)	85.7	84.9	84.2	82.5
Additionally allowed regions (%)	14.0	14.9	15.8	17.5
Generously allowed regions (%)	0.3	0.0	0.0	0.0
Disallowed regions (%)	0.0	0.1	0.0	0.0

† This value includes atoms in the multiple conformations of the succinate tail.
